# Impact of blue-collar vs. white-collar occupations on disease burden in psoriatic arthritis patients: A Swiss clinical quality management in rheumatic diseases cohort study

**DOI:** 10.1007/s10067-024-07077-1

**Published:** 2024-08-07

**Authors:** Nina Colla, Julia-Tatjana Maul, Enriqueta Vallejo-Yagüe, Andrea Michelle Burden, Burkhard Möller, Michael J. Nissen, Nikhil Yawalkar, Eleftherios Papagiannoulis, Oliver Distler, Adrian Ciurea, Raphael Micheroli

**Affiliations:** 1 Department of Rheumatology, University Hospital Zurich, University of Zurich, Schmelzbergstrasse 24, 8091 Zurich, Switzerland; 2 Department of Dermatology and Venerology, University Hospital Zurich, University of Zurich, Zurich, Switzerland; 3 Department of Health Sciences and Technology, ETH Zurich, Zurich, Switzerland; 4 Institute of Pharmaceutical Sciences, Department of Chemistry and Applied Biosciences, ETH Zurich, Zurich, Switzerland; 5 Institute of Primary Health Care (BIHAM), University of Bern, Bern, Switzerland; 6 Department of Rheumatology, Immunology and Allergology, University Hospital Inselspital Bern, Bern, Switzerland; 7 Department of Rheumatology, Geneva University Hospital, Geneva, Switzerland; 8 Department of Dermatology, University Hospital Inselspital Bern, Bern, Switzerland; 9 Statistics Group, SCQM Foundation, Zurich, Switzerland

**Keywords:** Manual vs. sedentary occupations, Psoriasis arthritis, Work disability

## Abstract

Biomechanical stress may exacerbate inflammation in psoriatic arthritis (PsA). This study aimed to investigate disease activity, work disability, and drug response/retention rates in PsA patients among two different occupation’s types: blue-collar workers (BCol) with manual labor versus white-collar workers (WCol) with sedentary occupations. PsA patients registered in the Swiss cohort (SCQM) were classified as BCol or WCol workers and assessed at the initiation of a biologic or targeted synthetic disease-modifying anti-rheumatic drug (b-/tsDMARD). We compared the baseline characteristics at treatment start and the DAS28-CRP for the 1-year remission. Treatment retention was investigated using Kaplan–Meier curves and Cox regression analysis. Multivariable models were adjusted for potential confounders. Of 564 patients, 29% were BCol, and 71% were WCol workers. Baseline disease activity was comparable between both groups. BCol workers were predominantly male (79.8%) and more work disabled at baseline (84.0% vs. 27.9%; *p* < 0.01). One hundred seventy-four treatment courses (TCs) of 165 PsA patients were included for longitudinal analysis. Occupation did not significantly influence the achievement of DAS28-CRP remission at 1 year. Kaplan–Meier analysis (*n* = 671) indicated longer retention for BCol workers (mean retention duration: 3.15 years vs. 2.15 years, *(p* = 0.006). However, adjusted Cox regression analysis did not corroborate these findings. This study indicates that physically demanding occupations correlate with increased rates of work disability among PsA patients, while treatment response seems to be unaffected by the patients’ occupation type. Additional research is required to thoroughly comprehend the relationship between physical workload, disease activity, and treatment outcomes.

**Table:** 

**Key Points** *• This study indicates that physically demanding occupations correlate with increased rates of work disability among PsA patients.* *• The treatment response among of PsA patients seems unaffected by the patients’ occupation type.*

**Supplementary Information:**

The online version contains supplementary material available at 10.1007/s10067-024-07077-1.

## Introduction

Psoriatic arthritis (PsA) is a chronic, clinically heterogeneous inflammatory rheumatic disease that belongs to the family of spondyloarthritides and primarily affects joints (arthritis) and entheses (enthesitis) [1, 2]. PsA patients often experience reduced quality of life and diminished work productivity [1, 3, 4]. Up to 40% of PsA patients experience work disability due to their disease, especially those with high disease activity [4]. This can be very costly, as sick leave and related expenses can add up quickly [5–7]. However, biological treatments have shown promise in improving these socioeconomic outcomes [8, 9].

The pathogenesis of PsA is the result of a multifaceted interaction between genetic, environmental, and unknown factors. One proposed environmental trigger for inflammation in PsA is biomechanical stress, sometimes called the “deep Köbner phenomenon” [10–12]. The relationship between high levels of biomechanical stress and joint damage has been investigated in a mouse model of spondyloarthritis [13]. Even on an immunology level, mechanical stress seems to have an important role in the pathogenesis of enthesis [14, 15]. Similarly, a study by Wervers et al. found that PsA patients who avoided physical activity experienced less enthesitis [16].

Occupations can be broadly categorized as either physically demanding (i.e., blue-collar (BCol) workers) or less demanding (i.e., white-collar (WCol) workers). Because BCol workers often face higher levels of biomechanical stress on their joints and entheses, this could trigger or worsen inflammation in these areas, leading to higher disease activity or poorer response to medication among PsA patients who work in physically demanding occupations.

Despite these hypotheses, the direct implication of work-related physical demands in PsA patients remains unexplored. It remains unclear whether PsA patients with high physical workloads experience increased disease activity or diminished treatment response. Therefore, this study aimed to compare disease activity, treatment effectiveness, and work disability among PsA patients with high (BCol workers) and low (WCol workers) physical workloads.

## Methods

### Study design and data source

This observational cohort study included patients with PsA from the Swiss Clinical Quality Management in Rheumatic Diseases (SCQM) registry from January 2000 to September 2020.

The SCQM registry includes patient characteristics and disease specific longitudinal data collected during routine rheumatology clinical practice and through patient-reported questionnaires — and only with their written informed consent.

The study included adult (≥ 18 years old) PsA patients from the SCQM cohort who were treated with biological/targeted synthetic disease modifying anti-rheumatic drugs (b/tsDMARD) and who had available data on standardized physical examination by their treating rheumatologist at the start of the new b/tsDMARD (i.e., baseline data) and at follow-up.

Patients were excluded if any of the following applied: missing information on BCol or WCol working sector; treated with off-label medications, overlap with another bDMARD treatment, or if there was no follow up data. Additionally, we excluded patients with an absence from work longer than 4 weeks during the observed time of 1 year and if the patient was work disabled at baseline. This last exclusion criterion was chosen because impact of work on the disease characteristics could not be drawn without sufficient exposure to their occupation. Work disability was defined as absent from work due to psoriatic arthritis and was assessed by their treating rheumatologist and recorded during the regular visits. Furthermore, we excluded TCs started in remission at baseline (DAS28-CRP < 2.6). The patients’ disposition is shown in Table 1.

**Table 1:**
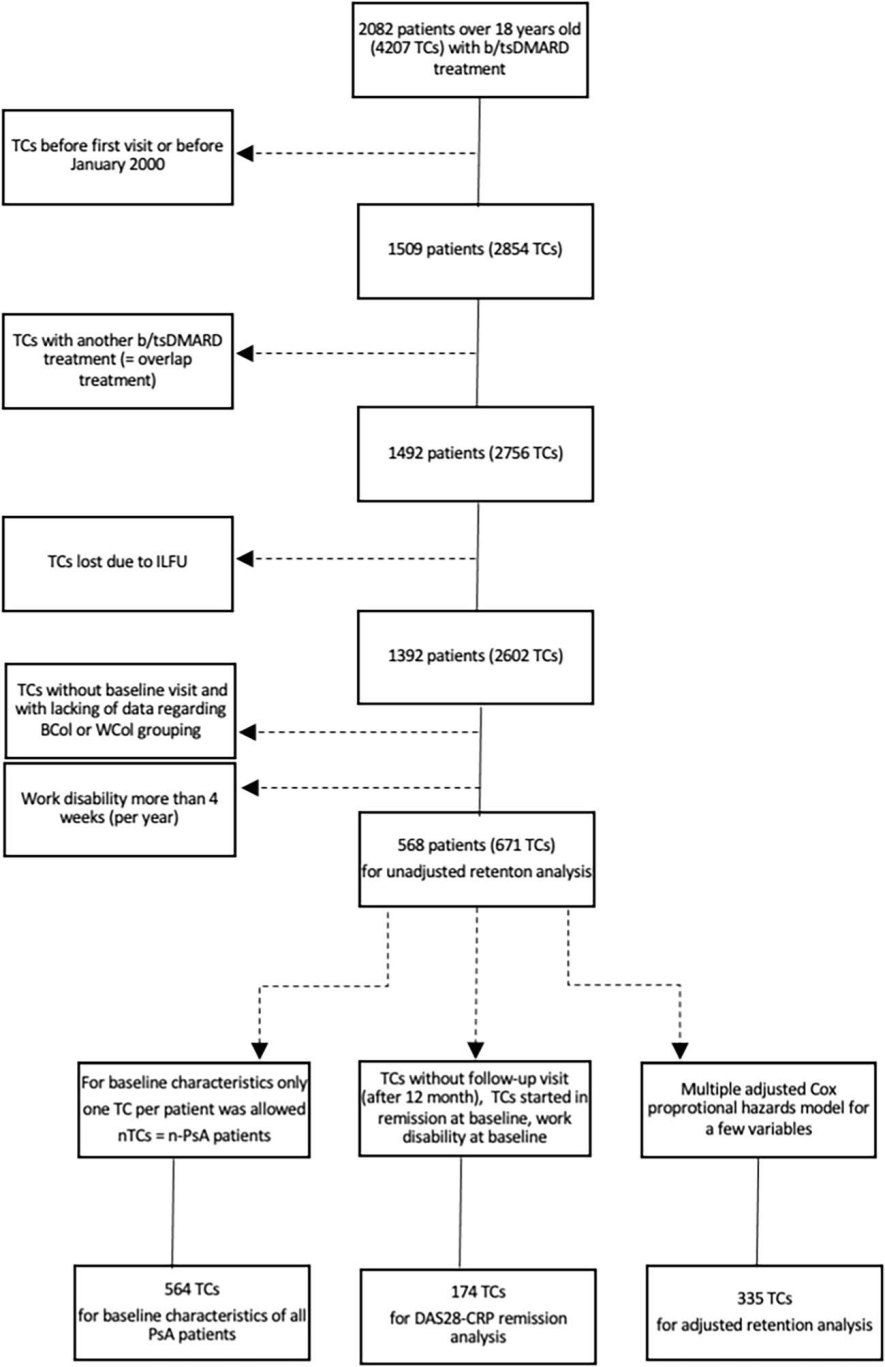
Patients disposition table of the study cohort: the last three rectangles showing (left side) the TCs for baseline, (middle) the TCs for 1 year DAS28-CRP remission, and (right side) the TCs for the adjusted retention analysis

*b/tsDMARDs* biologic or targeted synthetic disease-modifying anti-rheumatic drug (tumor necrosis factor inhibitors (TNFi) (i.e., adalimumab, etanercept, golimumab, certolizumab, and infliximab), biologics with other mode of action (i.e., secukinumab, ixekizumab, and ustekinumab), and the tsDMARD apremilast. Follow-up visit is defined as any visit between treatment start and the last recorded visit. *ILFU* “immediate loss to follow-up” corresponds to the cases where any further information expect baseline characteristics is missing, so that the baseline information and the follow-up data coincide. Overlap is defined as any length of simultaneous use of two or more of the b/tsDMARD drugs. *TC* treatment courses

The studied b/tsDMARDs included tumor necrosis factor inhibitors (TNFi) (i.e., adalimumab, etanercept, golimumab, certolizumab, and infliximab), as well as biologics with other mode of action (i.e., secukinumab, ixekizumab, and ustekinumab), and the tsDMARD apremilast. The tsDMARDs Janus Kinase inhibitors (JAKi) were excluded due to the limited number of treatment courses. The decision of which bDMARD a patient started was made by the treating rheumatologist in consultation with the patient.

To differentiate patients according the physical demand of their job, we decided to categorize into BCol and WCol jobs based on the official International standard Classification of Occupation (ISCO), as it been used in the European Foundation for the improvement of living and working conditions [17]. To determine whether a patient worked in a BCol or WCol sector, we used information from two routinely collected questions: one about the work sector (possible answers include: “Transportation,” “Manufacturing,” “Agriculture,” “Service,” “Housekeeper,” “Trainee” (e.g., Student) and “Other”), and another about the physical stress level at work (high vs. low work-related physical stress). In the supplement, you will find the corresponding questions. If the patient is reported working in “Transportation,” “Manufacturing,” or “Agriculture",” we classified them as BCol workers. If they are reported working in “Service,” we classified them as WCol workers. For patients who reported “Other” or a job category other than the abovementioned (such as “Housekeeper” or “Trainee”), we classified them as WCol if they reported low stress level or if the stress level value was missing; if they reported high stress level, we classified them as BCol. In cases where patients reported both “Service” and an abovementioned BCol job, we classified them as BCol if they reported high stress or if the stress level value was missing, and WCol if they reported low stress.

### Study exposure

Primary study exposure was the patients’ type of occupation, defined as BCol (manual labor; physically demanding) and WCol (sedentary or less physically demanding occupations).

### Study outcomes and follow-up

The primary question addressed the association between work-related high physical stress and various disease characteristics at baseline, focusing on disease activity and work disability.

Secondary outcomes were 1-year remission by DAS28-CRP (using disease activity score-28 (DAS28) and C-reactive protein (CRP) below 2.6), assessed at 12-month follow-up (± 90 days) and overall treatment retention of b-/tsDMARDs between the two groups using univariable and multivariable analyses.

Drug retention time of treatment courses (TCs) was defined as the time from the first dose (treatment start) until the last dose (treatment stop). Treatments that were not stopped at the end of the study were censored at the patient’s last database entry.

### Statistical analysis

Baseline patient characteristics were collected at the start of new b-/tsDMARD (within a range of − 90 and + 30 days from start). Baseline information included patient demographics (e.g., age, sex, and BMI), disease characteristics and disease activity, type of treatment, and self-reported information on the patients’ sports activity in their leisure time, work disability, work quitting, and invalidity pension. The respective detailed questions are provided in the supplement.

Baseline patient characteristics were described overall and stratified by their occupational sector as BCol or WCol and were compared using Fisher’s exact test for categorical variables, and Wilcoxon-Kruskal test for continuous variables. For baseline characteristics, we only considered the first TC per patient.

For the longitudinal analysis (DAS28-CRP remission and retention rates) multiple TCs per patient were possible. To account for the correlation of multiple observations per patient, a generalized estimating equation (GEE) model was used to analyze potential differences in 1-year remission between the BCol and the WCol groups.

We included the following variables as potential confounders for the relationship between worker status and the treatment effectiveness outcomes: sex and disease duration (years). Body mass index (BMI) category according to WHO [18], baseline disease activity (DAS28-CRP), enthesitis at baseline, type and line of treatment, and co-therapy with conventional synthetic DMARD (csDMARD) or steroids were included in the model as explanatory variables for reaching DAS28-CRP remission.

Sex was a dichotomous category (female; male). BMI categories included normal weight (BMI < 25 kg/m^2^), overweight (BMI 25 to 30 kg/m^2^), and obese (BMI > 30 kg/m^2^). Enthesitis was a dichotomous category (yes; no) based on the Maastrich Ankylosing Spondylitis Enthesitis Score (MASES). Type of treatment was defined as TNFi, biologic with other mode of action (OMA), and tsDMARD. Line of treatment included 1st line, 2nd line, and ≥ 3rd line. Co-therapy with csDMARD was defined as the use of csDMARD or steroids at the time of the bDMARD initiation.

Treatment retention was compared between the BCol and the WCol groups, first, using Kaplan–Meier survival curves with log-rank tests, and second, using Cox (proportional hazard) regression with the inclusion of the potential confounders sex and disease duration (years) and further variables as explanatory variables for drug retention time: body mass index (BMI) category, baseline disease activity (DAS28-CRP), type and line of treatment, and co-therapy with conventional synthetic DMARD (csDMARD) or steroids. There were too little data for retention regarding enthesitis, in addition we looked at typical confounders such as smoker status and physical activity per week. Smoker status was a dichotomous category (yes; no) and physical activity per week included no sport, less than 1 h per week, between 1 and 2 h per week or more than 2 h per week. Additionally, as sensitivity analysis, this was repeated without the exclusion of those with > 4 weeks of work disability.

All analyses were performed using R statistical software (R version 4.1.2 (2021–11-01).

### Ethical considerations

All included patients were over 18 years old and provided informed consent before inclusion into the SCQM. The study was approved by the Ethics Committee of the Canton of Zuerich (BASEC 2022–00272).

## Results

### Baseline characteristics

The study included 564 patients, with 29% (*n* = 168) classified as BCol workers and 71% (*n* = 396) as WCol workers. Table 2 provides an overview of these patients’ baseline characteristics. Compared to WCol workers, BCol workers were more often men (79.8% vs. 41.7%; *p* < 0.01), experienced higher rates of work disability (84.0% vs. 27.9%; *p* < 0.01), and were more likely to quit their jobs due to PsA (19.1% vs. 7.8%; *p* = 0.03). DAS28-CRP and patient-reported outcomes (HAQ, EQ-5D, SF 12-PCS, and SF 12-MCS) were similar between the groups. TNFi treatment was the most frequent biological treatment (88.1% in BCol; 88.9% in WCol), followed by biologics with other modes of action (6.0% in BCol; 7.3% in WCol) and tsDMARD (6.0% in BCol; 3.8% in WCol). Initiation in co-therapy was less frequent in BCol than in WCol workers 26.2% (*n* = 77) and 73.8% (*n* = 217), respectively. In total, 69.4% (*n* = 204) completed the csDMARD co-therapy, with 65% in BCol workers (*n* = 50) and 71% (*n* = 154) in WCol workers. There was no statistically significant difference regarding physical activity at leisure time but a tendency for less physical activity in the BCol group.

**Table 2:** Baseline characteristics at the start of first b-/tsDMARD

Variable		Total	Work sector		*p-value*
			BCol workers	WCol workers	
		*n* = 564	*n* = 168	*n* = 396	
*Patient characteristics*					
Male sex, %	564	53	79.8	41.7	< 0.01*
Age, mean years (*SD*)	564	46 (11)	45 (13)	47 (11)	0.06
Disease duration, mean years (*SD*)	548	9 (9)	8 (7)	9 (9)	0.35
Current smokers, %	371	22.6	28	24.5	0.1
BMI (kg/m^2^), %	564				0.93
< 25	189	33.5	32.7	33.8	
25–30	258	45.7	47	45.2	
> 30	117	20.7	20.2	21	
*Disease characteristics*					
IBD, %	473	3.6	3.6	3.6	1
Dactylitis ever, %	564	55.1	57.7	54	0.46
Enthesitis ever, %	564	65.2	63.7	65.9	0.63
Nail involvement ever, %	564	21.1	17.9	22.5	0.26
Severe skin involvement, %	518	10.2	14	8.7	0.08
Uveitis, %	564	2	1	1	0.51
*Disease activity*					
DAS28-CRP, mean (*SD*)	473	3.2 (1.1)	3.3 (1.1)	3.2 (1.1)	0.37
MASES, median (*IQR*)	375	1 (3)	0 (2)	1 (3)	0.49
HAQ, median (*IQR*)	550	0.5 (0.8)	0.5 (0.9)	0.5 (0.6)	0.85
EQ-5D, mean (*SD*)	371	66.1 (18.7)	65.1 (22.5)	66.5 (17.1)	0.74
SF12-PCS, mean (*SD*)	544	45.9 (11.2)	44.7 (11.5)	46.4 (11)	0.11
SF12-MCS, mean (*SD*)	544	39.9 (9.8)	40 (10.8)	39.8 (9.7)	0.83
*Physically activity*					
All patients	358		98	260	0.35
No physically activity per week	358	120	39	81	
Physically activity < 1 h/week	358	74	19	55	
Physically activity 1–2 h/week	358	105	23	82	
Physically activity > 2 h/week	358	59	17	42	
*Work disability (due to PsA as reported by patients)*					
Work disability, %	93	43	84	27.9	< 0.01*
Job quitting ever, %	216	11.1	19.1	7.8	0.03*
Application of invalidity pension, %	169	12.4	23.3	10.1	0.06
*Treatment*					
All DMARD	564				0.44
TNFi, %	500	88.7	88.1	88.9	
OMA, %	39	6.9	6	7.3	
sDMARD, %	25	4.4	6	3.8	
csDMARD co-therapy at baseline	564	294	77	217	0.05
Completed csDMARD co-therapy	564	204	50	154	0.04

*BMI* body mass index, *CRP* C-reactive protein, *csDMARD* co-therapy: use of csDMARD or steroids at the time of the bDMARD initation, *DAS* disease activity score, *DMARD* disease-modifying anti-rheumatic drug, *EQ-5D* European Quality of Life 5 -domains, *HAQ* health assessment questionnaire, *IBD* inflammatory bowel disease, *MASES* Maastricht Ankylosing Spondylitis Entheses Score, others = *OMA*, other modes of action than inhibition of TNF, *PsA* psoriatic arthritis, *SF12-MCS* mental component score of the short form questionnaire with 12 questions, *SF12-PCS* physical component score of the short form questionnaire with 12 questions, “severe” as reported by the physician, *TNF* tumor necrosis factor inhibitor, *tsDMARD* targeted synthetic disease-modifying anti-rheumatic drug

^*^ Significance levels of two groups when *p*-value < 0.05

### Response rate

For the 1-year remission rate by DAS28-CRP, 174 TCs (of 165 patients) were analyzed. Of the 174 TCs, 103 achieved remission (69% in WCOl group (*n* = 71) and 31% in BCol group (*n* = 32). BCol/WCol status was not associated with differences in achieving DAS28-CRP remission after 1 year: 60% in WCol group (95% *CI* from 0.51 to 0.69) vs. 57% in BCol group (95% *CI* from 0.44 to 0.70).

In the adjusted GEE analysis in Table 3, lower remission rates were significantly associated with two factors: female sex (*OR* 0.31, 95% *CI* 0.14–0.66,* p* = 0.002) and BMI > 30 kg/m^2^ (*OR* 0.30, 95% *CI* 0.13–0.68, *p* = 0.004), but BCol/WCol status did not show an association. Disease duration did not impact the DAS28-CRP remission rate after 1 year of treatment. The number of previous treatments showed a large confidence interval (second line: *OR* 0.57, 95% *CI* 0.17–1.9,* p* = 0.363 and third line: *OR* 0.77, 95% *CI* 0.26–2.31,* p* = 0.646) with a tendency to lower remission rate. Patients without enthesitis at baseline (*OR* 2.09, 95% *CI* 1.03–4.25,* p* < 0.042) showed a higher remission rate.

**Table 3:** Generalized estimating equation model for DAS28-CRP remission in BCol workers and WCol workers at 1 year of treatment

Variable	*OR*	95% *Cl*	*p-value*
BCol workers (ref: WCol workers)	0.59	0.27–1.30	0.191
DAS28-CRP	0.65	0.42–1.01	0.058
DMARD type OMA (ref: TNFi)	0.86	0.14–5.14	0.869
DMARD type tsDMARD (ref: TNFi)	0.82	0.24–2.79	0.753
Female sex	0.31	0.14–0.66	0.002*
Disease duration	0.98	0.95–1.02	0.391
DMARD line 2nd (ref: 1st line)	0.57	0.17–1.90	0.363
DMARD line > = 3rd (ref: 1st line)	0.77	0.26–2.31	0.646
csDMARD co-therapy no vs. yes	1.12	0.52–2.40	0.777
BMI 25–30 (ref: BMI < 25)	0.60	0.26–1.38	0.228
BMI > 30 (ref: BMI < 25)	0.30	0.13–0.68	0.004*
No enthesitis (ref: enthesitis at baseline)	2.09	1.03–4.25	0.042*

Analysis performed with 174 TCs of 165 patients

*BMI* body mass index, *CRP* C-reactive protein, *csDMARD* conventional synthetic disease-modifying anti-rheumatic drug, *csDMARD* co-therapy: use of csDMARD or steroids at the time of the bDMARD initation, *DAS* disease activity score, *DAS28-CRP* disease activity score (28 joints), *DMARD* disease-modifying anti-rheumatic drug, *OMA* other modes of action, *TNFi* tumor necrosis factor inhibitor, *tsDMARD* targeted synthetic disease-modifying anti-rheumatic drug

^*^ significance levels of two groups when *p*-value < 0.05

### Retention rate

**Fig. 1:**
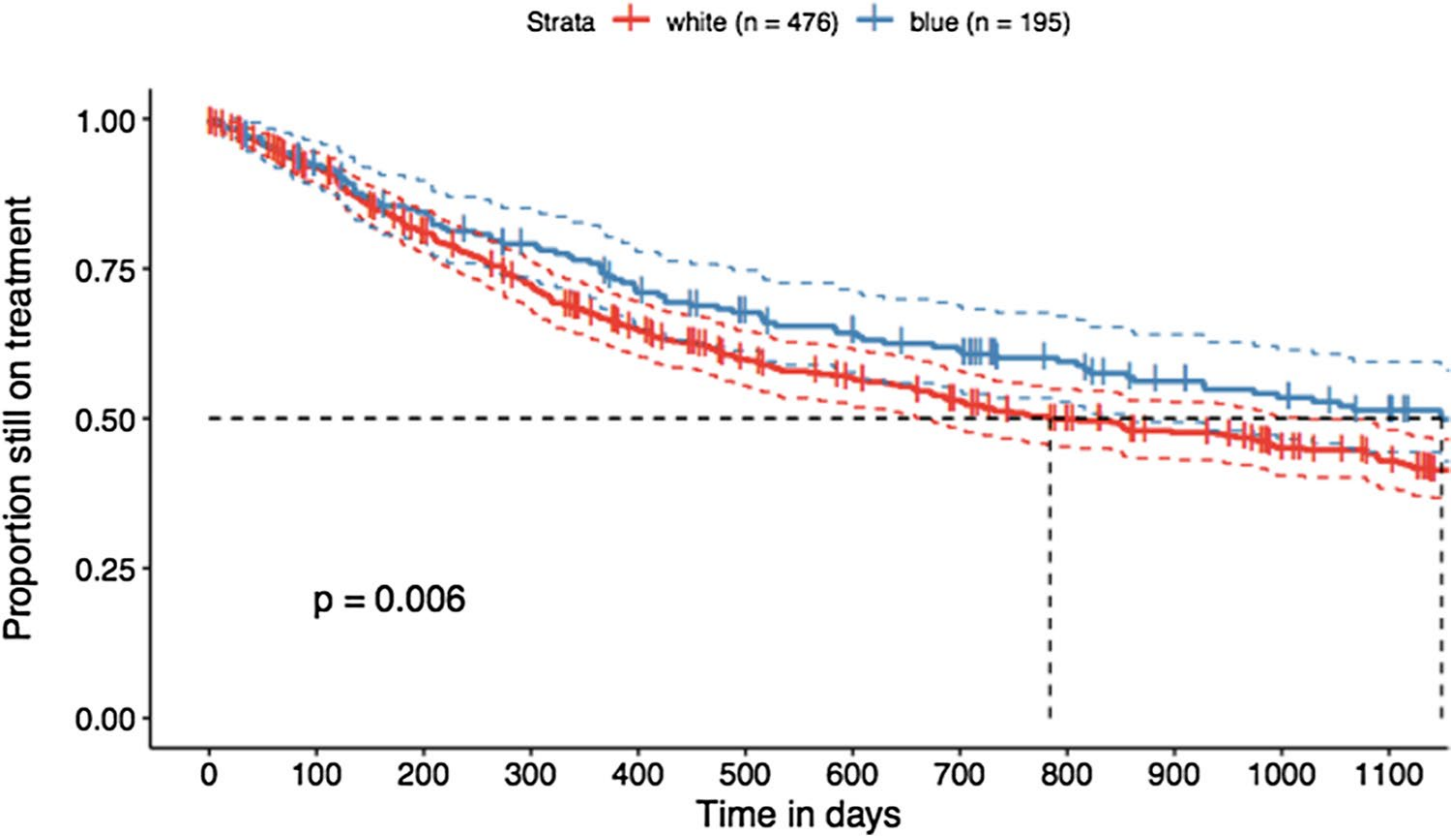
Kaplan–Meier plot (excluding patients with more than 4 weeks of work disability). Kaplan–Meier plot for treatment retention of 671 TCs (BCol: 195 and WCol: 476); blue, blue-collar (BCol) workers; red, white-collar (WCol) workers; *p*-value = 0.006, median retention duration in BCol workers: 3.15 years (1149 days) in WCol workers: 2.15 years (784 days)

Analyses of the retention rate included 671 TCs (195 TCs for BCol and 476 TCs for WCol). The Kaplan–Meier plots in Fig. 1 show that BCol workers have significantly longer median retention duration than WCol workers (3.15 years (1149 days) vs. 2.15 years (784 days), log-rank test, *p* = 0.006).

**Fig. 2:**
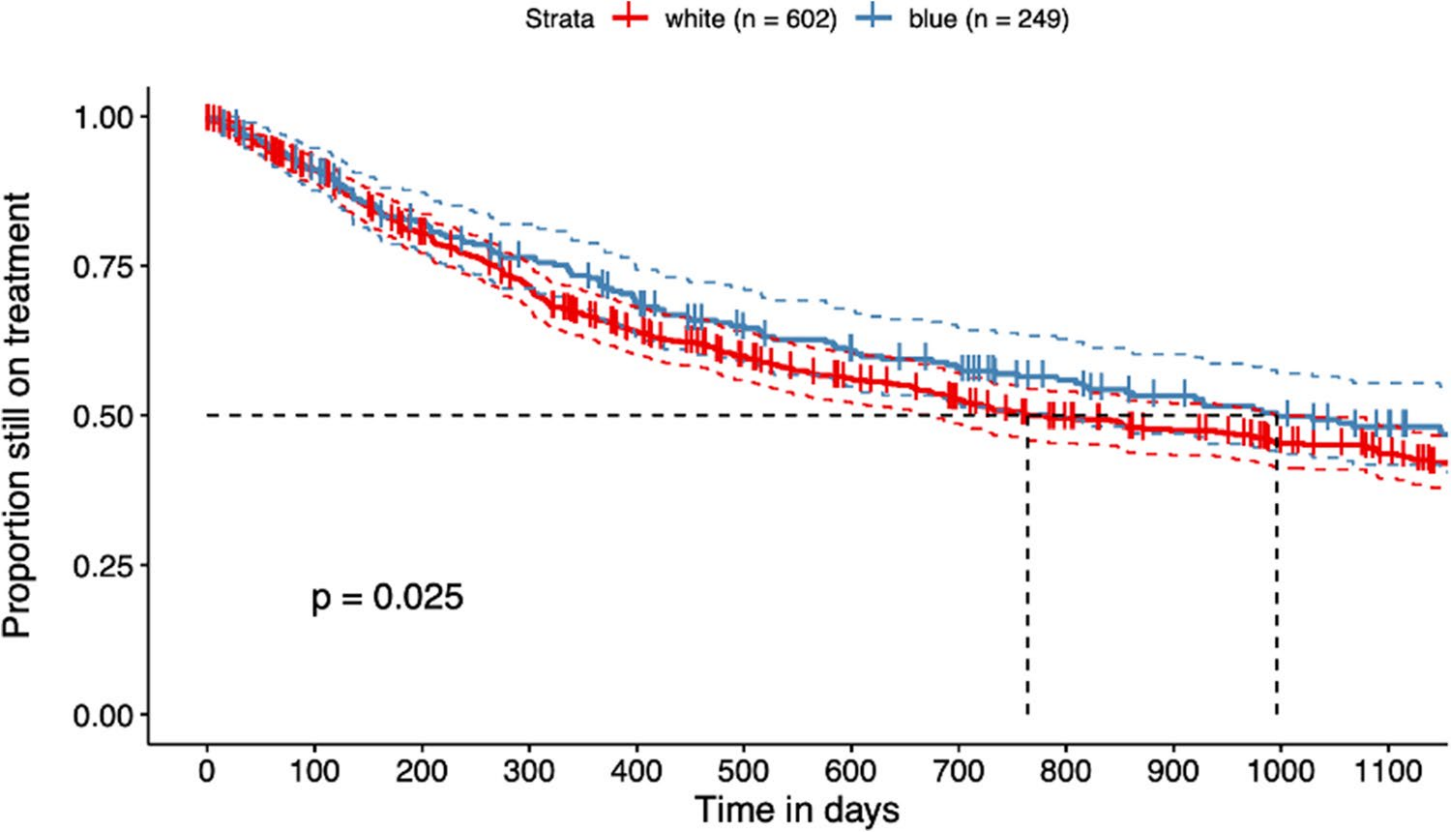
Kaplan–Meier plot (all included PsA patients independent of reported work disability). Kaplan–Meier plot for treatment retention of 851 TCs (BCol: 249 and WCol: 602); blue, blue-collar (BCol) workers; red, white-collar (WCol) workers; *p*-value = 0.025, median retention duration in BCol workers: 2.10 years (996 days), in WCol workers: 2.73 years (764 days)

In a sensitivity analysis that included all PsA patients (*n* = 851; 249 BCol workers, 602 WCol workers), even those who are absent from work for more than 4 weeks per year, the Kaplan–Meier plots in Fig. 2 show a significantly longer median retention duration in BCol workers compared to WCol workers (2.10 years (996 days) vs. 2.73 years (764 days), log-rank test,* p* = 0.025).

The multiple adjusted retention analysis performed on 335 patients with PsA (92 BCol workers and 243 WCol workers) does not confirm the difference in Kaplan-Meyer plots (*HR* 0.71; 95% *CI *0.48–1.05; *p* = 0.086), as shown in Table 4. Female sex was the only variable associated with significantly shorter retention (*HR* 1.91; 95% *CI* 1.36–2.66; *p* < 0.001). Regarding physical activity, there was a tendency, that the more physical activity a patient performed, the higher was the retention rate. But this was not statistically significant. Regarding csDMARD co-therapy and regarding smoking status, there was no difference between the two groups.

**Table 4:** Multiple adjusted Cox proportional hazards model for retention rate analysis

Variable	*HR*	95% *Cl*	*p-value*
BCol workers (ref: WCol workers	0.71	0.48–1.05	0.086
Female sex	1.91	1.36–2.66	< 0.001*
DMARD type OMA (ref: TNFi)	0.86	0.49–1.49	0.582
DMARD type tsDMARD (ref: TNFi)	1.35	0.81–2.25	0.248
DMARD line 2nd (ref: 1st line)	1.06	0.77–1.47	0.707
DMARD line > = 3rd (ref: 1st line)	1.12	0.74–1.71	0.589
csDMARD co-therapy no vs. yes	1.11	0.81–1.53	0.513
BMI 25–30 (ref: BMI < 25)	1.13	0.82–1.56	0.464
BMI > 30 (ref: BMI < 25)	0.89	0.60–1.33	0.573
No smoker (ref: smoker)	1.32	0.90–1.93	0.160
Physically activity < 1 h/week (ref: no)	0.93	0.62–1.40	0.722
Physically activity 1–2 h/week (ref: no)	0.73	0.50–1.05	0.086
Physically activity > 2 h/week (ref: no)	0.94	0.63–1.41	0.769

Analysis performed with 335 TCs; BCol 92 TCs and WCol 243 TCs

*BMI* body mass index, *csDMARD* conventional synthetic disease-modifying anti-rheumatic drug, *csDMARD* co-therapy: use of csDMARD or steroids at the time of the bDMARD initation, *DMARD* disease-modifying anti-rheumatic drug, *OMA* other modes of action, *TNFi* rumor necrosis factor inhibitor, and *tsDMARD* targeted synthetic disease-modifying antirheumatic drug

^*^ significance levels of two groups when *p*-value < 0.05

## Discussion

This study showed that PsA patients in the BCol group were predominantly male and reported significantly higher work disability than PsA patients in the WCol group. No significant differences in response or retention rates for the various b-/tsDMARD treatments were observed between BCol and WCol workers in PsA.

Work disability is a complex issue that can be influenced by multiple factors, especially psychosocial and physical health, socioeconomic status and the activity of the inflammatory disease itself [19–26]. In this study, the higher work disability observed among PsA patients — especially men — in physically demanding occupations (i.e., BCol workers) can potentially be attributed to several factors. First and regardless of PsA, the higher levels of physical stress associated with these jobs, which are often monotone and repetitively, can lead to higher work disability due to musculoskeletal disorders or injuries and early osteoarthritis [19–22].

Second, BCol workers may additionally face greater socioeconomic challenges and often are less educated than WCol workers [24]. This can further impact their ability to manage their condition and remain employed [25]. Barlow et al. showed that work disability was associated with lower education status, comorbidities and higher physical impairment in patients with ankylosing spondylarthritis [26].

It is worth noting that other factors, such as comorbidities and lifestyle factors, may also contribute to the higher work disability [26, 27]. Patients in everyday clinical practice should be educated about their disease and comorbidities and the risks of becoming work disabled. Several studies showed that even short time work disability is associated with higher rates of further work disabilities and even with higher mortality rate [28–31]. Early intervention and work retraining might help prevent long-term work disability and maintain employment, one of the most fundamental social determinants of health [26, 32].

In adjusted analysis we showed no significant differences in response or retention rates for the various b-/tsDMARD treatments between BCol and WCol workers in PsA. In the unadjusted analysis for the retention rate, PsA patients showed a significant longer retention rate in BCol workers — regardless of whether the patients were often absent from work or not, so that the amount and type of physical workload may not have that impact on response or retention rates. Moreover, we postulate that WCol workers — with more educated patients and more women — may have higher expectations on the therapy and want to be more involved in their therapy than BCol workers [33]. Finckh et al. showed in a comparative study of patients with rheumatoid arthritis in different European countries that the socioeconomic status of the country (high gross domestic product (GDP) was inversely proportional to the retention rate of the bDMARDs [34].

The primary limitation of this study is the observational nature of the data, which makes it challenging to establish an association between physical workload and disease burden. More specifically, the lack of significant difference in disease activity at baseline between BCol and WCol patients could be due to the higher prevalence of work disability among BCol workers. In other words, BCol workers may be experiencing higher levels of inflammation while actively working, which eventually self-regulates through a reduced ability to work. Therefore, it is not necessarily accurate to assume that BCol workers do not have higher disease activity than those with less physically demanding jobs. Instead, it may be more accurate to conclude that low physical workload does not necessarily lead to higher disease activity when compared to individuals who are unemployed and have a history of working in physically demanding jobs.

Moreover, the longitudinal analysis — although excluding patients who did not work for more than 4 weeks — is additionally complicated because some patients in the BCol group may be retrained or working in adapted occupations, which can impact their physical workload and thus affect the interpretation of the results.

One source of weakness in the study is that patients will not always be correctly allocated to either the BCol- or WCol workers. Furthermore, the workload within each of the two groups (BCol and WCol workers) may vary considerably, thus possibly leading to distorted results.

In conclusion, this study suggests that physically demanding occupations correlate with increased rates of work disability among PsA patients, particularly men. Further research is needed to fully understand the impact of physical workload on disease burden and to develop effective interventions for preventing long-term work disability among PsA patients.

## Supplementary Information

Below is the link to the electronic supplementary material.

Supplementary file1 (DOCX 17 KB)
