# Supplementary material for: Impact of blue-collar vs. white-collar occupations on disease burden in psoriatic arthritis patients: A Swiss clinical quality management in rheumatic diseases cohort study
Source: Clin Rheumatol. 2024 Aug 7;43(10):3147–55. doi: 10.1007/s10067-024-07077-1 (PMC11442542; doi:10.1007/s10067-024-07077-1)
Supplement: Supplementary file 1 — Supplementary file1 (DOCX 17 KB) [file 10067_2024_7077_MOESM1_ESM.docx]

**Supplementary information**

**Supplement**

**1. Selected questions from the SCQM questionnaires**

Questions about type of occupation

- “In which occupations are you now or were you last employed?

Multiple answers were possible out of:

“Transportation”, “Manufacturing”, “Agriculture”, “Service”, “Housewife”, “Trainee” (e.g., Student) and “Other”

- "What is the physical stress level of your occupation?"

Possible answers were high or low.

Questions about physical activity in leisure time:

- “How much time do you typically spend walking, biking, or similar outdoor activities each day?”

Possible answers were: no time, less than 30 minutes, between 30 - 60 minutes, or over an hour.

- “How long do you exercise (or do any physical activity during leisure time) so that you get sweaty and out of breath?”

Possible answers were: no sport, less than 1 hour per week, between 1 - 2 hours per week or more than 2 hours per week.

Question about work ability

- "Are you currently incapacitated for work due to the rheumatological disease?"

Answer was possible with either yes or no

- "Did you have one or more absences from work due to your rheumatological disease during the last 12 months?”

Answer was possible with either yes or no, and if yes, how long in total?

Possible answers with either “up to four weeks (during the last 12 months)” or “more than four weeks (during the last 12 months)”
